# Double Cortex Syndrome (Subcortical Band Heterotopia): A Case Report

**Published:** 2015

**Authors:** Ali Akbar Momen, Mehdi Momen

**Affiliations:** 1 MusculoSkeletal Rehabilitation Research Center, Ahvaz Jundishpour University of Medical Sciences, Ahvaz, Iran; 2 Child Neurologist, Pediatric Department , Ahvaz Jundishpour University of Medical Sciences, Ahvaz, Iran; 3 Ahvaz Jundishpour University of Medical Sciences, Ahvaz, Iran

**Keywords:** Developmental delay, Seizures, Band heterotopia of brain

## Abstract

**Objective**

Approximately 5–10% of preschool age children are considered developmentally disabled. Brain Magnetic Resonance Imaging (MRI) plays a key role in the diagnostic evaluation in these children. Many congenital or acquired brain anomalies are revealed with MRIs. Although the majority of these abnormalities are sporadic but patients with subcortical band heterotopia or double cortex syndrome have sex-linked inheritance. We are going to present the first case in Iran from Ahvaz city, which was presented with status epilepticus associated with developmental delay and finally diagnosed as double cortex syndrome, because band heterotopia cases especially for continuous or generalized form is rare. A 4.5-year-old developmentally delayed girl was admitted for generalized tonic clonic seizure attack of 1 hr, upward gaze, locked mouth, and urinary incontinence (status epilepticus) in the child neurology ward. She had a history of recurrent seizures that started as febrile seizures since she was 12 months of age and had frequent admissions for having recurrent seizure attacks. She was the only child of consanguineous parents with negative family history of any neurologic problems. She was a product of uneventful term pregnancy, vaginal delivery with a low Apgar score at birth who was admitted for six days in the neonatal ward for hypotonia and cyanosis. At 4.5 years of age, she had HC: 45cm (<3%) Length: 102 cm (25–75%), and BW: 18kg (75%). She was able to sit, walk with support, speak a few words, and communicate with others. A physical exam was unremarkable. Lab data including CBC, blood biochemical, and urinalysis results were all within normal limits, but the electroencephalography (EEG) revealed generalized poly spike-wave discharges. A brain MRI showed corpus callosal dysplasia, generalized band heterotopia, and polymicrogyria. She was discharged home with oral valproate and regular outpatient follow-ups. In the diagnostic evaluation of developmentally delayed and epileptic children, a brain MRI is strongly recommended for accurate diagnosis of anomalies such as neuronal migration disorders (band heterotopia) and others, because appropriate therapeutic management, prognosis, prevention, and genetic counseling for prenatal diagnosis are dependent on definite diagnosis of the proband case.

## Introduction

The cerebral cortex is composed of multiple neuronal types and this complex organization is crucial for cognitive functions that define us as human (1). Subcortical band heterotopia (SBH) has a sex-linked inheritance and more than 90% of the affected individuals are females. The rare possibility of an affected male is the result of somatic mosaicism or hemizygous mutations for DCX gene (2). J. Ono, et al has reported a 7-yearold boy with double cortex syndrome(3). Mutations of the DCX gene are the most common identified cause in female patients with SBH (4). Affected individuals typically present with epilepsy and a variable degrees of mental retardation. Seizures often start in the first decade of life. They may progress to multiple seizure types and are usually refractory to medication (5). Neurological examination may be normal, but dysarthria, hypotonia, poor fine motor control, or, rarely, a pyramidal syndrome may be present (6). The mainstay of diagnosis is the MRI, which shows the characteristic isointensity of the heterotopic band with the cortex in all imaging sequences. The band varies in size and thickness, the appearance of the overlying cortical mantle on MRI may be normal, or it may demonstrate a spectrum from agyria to pachygyria (6). We are going to present the first case in Iran from the city of Ahvaz, who was admitted to a pediatric neurology ward with status epilepticus and finally diagnosed as double cortex syndrome.

## Case report

**Fig 1:**
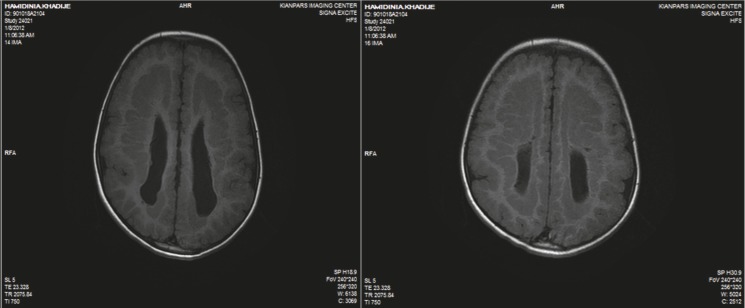
Axial T1-weighted brain MRI shows polymicrogyria, complete band heterotopia, and colpocephaly

**Fig 2:**
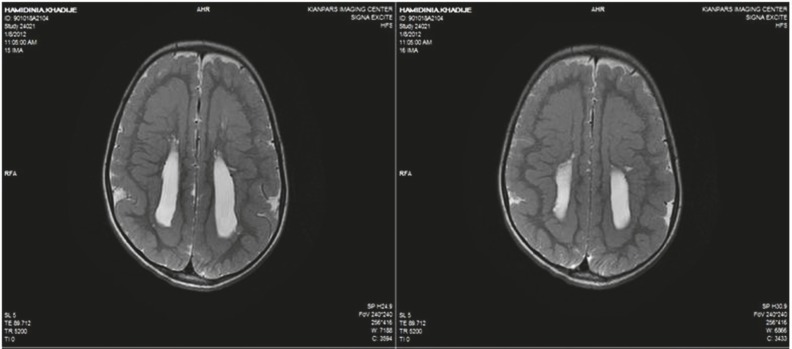
Axial T2-weighted brain MRI shows polymicrogyria, complete band heterotopia, and colpocephaly

**Fig 3:**
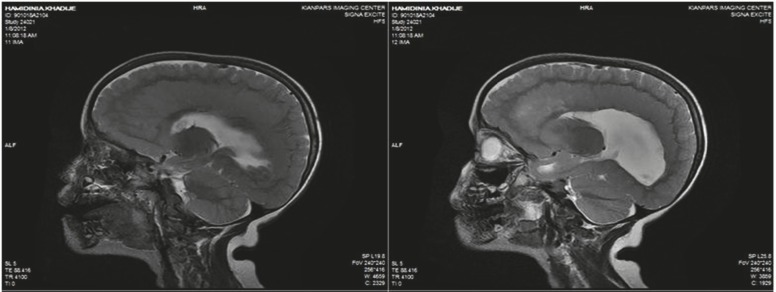
Sagittal T2-weighted brain MRI, shows polymicrogyria, complete band heterotopia

**Fig 4:**
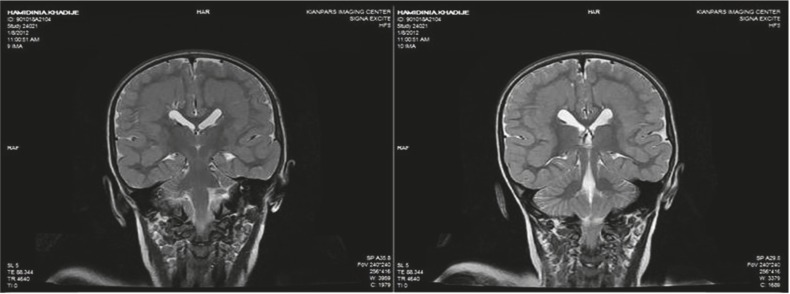
Coronal T2-weighted brain MRI, shows polymicrogyria, complete band heterotopia, and corpus callosal dysplasia

The patient is a 4.5-year-old girl with a known case of symptomatic epilepsy and global developmental delay, presenting with status epilepticus. She had general tonicclonic movements, upward gaze, and jaw lock followed by urinary incontinency when admitted Seizures lasted for 1 h after which she entered a postictal phase with drowsiness. She was born at term via a vaginal delivery after an uncomplicated pregnancy. Due to hypotonia, cyanosis, and a low Apgar score she was admitted to the neonatal ward and was discharged after 6 days without any complications. She was admitted to the hospital several times for controlling seizures. Her seizures started with concomitant fever when she was 1 year old. Seizures were treated for 2 months, after which, treatment was stopped by parents due to low compliance. The parents did not remember the name of the antiepileptic drugs. She was the only child of consanguineous parents. There was no specific disease history in her family. She had normal growth but abnormal development. Her speech was limited to a few words. She sat alone and walked with assistance. A general examination revealed an oriented but agitated child who opened her eyes with stimulation, talked to the parents, and obeyed simple commands. She had a weight of 18 kg (75%), height of 102 cm (25–75%), and a head circumference of 45 cm (<3%). Motor and sensory evaluation was unremarkable. Blood biochemicals were in normal reference ranges. CBC showed leukocytosis (23900/µL) with 73% neutrophils. An EEG indicated generalized polyspike waves. On her last admission, a brain MRI was obtained and revealed corpus callosal dysplasia, polymicrogyria, and SBH (figures 1–4). She was discharged home with sodium valproate and asked for regular follow-ups at OPD.

and corpus callosal dysplasia.

## Discussion

Neuronal migration is essential for proper cortical functioning and neuronal migration disorders are an important cause of cortical malformations (7). More than 25 neuronal migration disorders result in death or improper positioning of the cortical neurons have been described in humans (8). Using an MRI, evaluation of developmental and neurological disorders has become easier than before (9). Neuronal migration disorders such as lissencephaly and SBH may have a wide variety of clinical manifestations, but developmental delays and epilepsy syndromes are usually the presenting symptoms. A. Palmini et al have presented 10 girls with mental retardation and seizures. Their clinical, radiologic, electrographic, neuropsychological, and therapeutic data were described (10). J. Ono, et al presented a moderately mentally retarded Japanese boy with normal male karyotype, 46, XY with SBH or double cortex syndrome (11). Several cases have been presented in literature. We are presenting the first case of SBH or double cortex from the city of Ahvaz, Iran. As described in our case, a patient with developmental delay and symptomatic epilepsy was treated with antiepileptic medications without any specific diagnosis, and the result was non-adherence to therapy by parents due to lack of information provided to them. By using an MRI in this patient, a specific diagnosis was made, information regarding prognosis and follow up was provided for parents, and as a result, her seizures were more effectively controlled.

**In conclusion**, double cortex syndrome is a serious neurological disorder. Working as physicians our duty is to reduce the burden for the family. The physician needs to judge whether to use imaging studies to make a diagnosis. The proper use of imaging modalities, unlike in the management of the above case, would lead to a diagnosis, and as a result, a management plan is implemented. This plan would help the patient and family as well as offer great benefits on preventing such syndromes from happening in future offspring by the use of prenatal testing and genetic counseling.
